# Immunomodulators in patients receiving extracorporeal membrane oxygenation for COVID-19: a propensity-score adjusted analysis of the ELSO registry

**DOI:** 10.1186/s13613-024-01368-1

**Published:** 2024-08-20

**Authors:** Ryan Ruiyang Ling, Kollengode Ramanathan, Liang Shen, Ryan P. Barbaro, Kiran Shekar, Daniel Brodie, Graeme MacLaren

**Affiliations:** 1 Yong Loo Lin School of Medicine, National University of Singapore, National University Health System, Level 9, 1E Kent Ridge Road, Singapore, 119228 Singapore; 2 Cardiothoracic Intensive Care Unit, National University Heart Centre, National University Hospital, National University Health System, Singapore, Singapore; 3 Biostatistics Unit, Yong Loo Lin School of Medicine, National University of Singapore, National University Health System, Singapore, Singapore; 4 Division of Paediatrics Critical Care Medicine, University of Michigan, Ann Arbor, USA; 5 Child Health Evaluation and Research Center, University of Michigan, Ann Arbor, MI USA; 6 Adult Intensive Care Services, Prince Charles Hospital, Brisbane, QLD Australia; 7 Queensland University of Technology, Brisbane, Australia; 8 University of Queensland, Brisbane and Bond University, Gold Coast, QLD Australia; 9 Division of Pulmonary and Critical Care Medicine, Department of Medicine, The Johns Hopkins University School of Medicine, Baltimore, MD USA

**Keywords:** Extracorporeal membrane oxygenation, Coronavirus disease 2019, Immunomodulatory treatment, Corticosteroids

## Abstract

**Background:**

Mortality for patients receiving extracorporeal membrane oxygenation (ECMO) for COVID-19 increased over the course of the pandemic. We investigated the association between immunomodulators and mortality for patients receiving ECMO for COVID-19.

**Methods:**

We retrospectively analysed the Extracorporeal Life Support Organisation registry from 1 January, 2020, through 31 December, 2021, to compare the outcomes of patients who received no immunomodulators, only corticosteroids, only other immunomodulators (selective interleukin blockers, janus-kinase inhibitors, convalescent plasma, and intravenous immunoglobulin), and a combination of corticosteroids and other immunomodulators administered either before or during ECMO. We used Cox regression models to estimate survival time until 90 days. We estimated the propensity score of receiving different immunomodulators using multinomial regression, and incorporated these scores into the regression models.

**Results:**

We included 7181 patients in the final analysis; 6169 patients received immunomodulators either before or during ECMO. The 90-day survival was 58.1% (95%-CI 55.1–61.2%) for patients receiving no immunomodulators, 50.7% (95%-CI 49.0–52.5%) for those receiving only corticosteroids, 62.2% (95%-CI 57.4–67.0%) for those receiving other immunomodulators, and 48.5% (95%-CI 46.7–50.4%) for those receiving corticosteroids and other immunomodulators. Compared to patients without immunomodulators, patients receiving either corticosteroids alone (HR: 1.13, 95%-CI 1.01–1.28) or with other immunomodulators (HR: 1.21, 95%-CI: 1.07–1.54) had significantly shorter survival time, while patients receiving only other immunomodulators had significantly longer survival time (HR: 0.79, 95%-CI: 0.66–0.96). The receipt of immunomodulators (across all three groups) was associated with an increase in secondary infections.

**Conclusions:**

In this cohort study, we found that immunomodulators, in particular corticosteroids, were associated with significantly higher mortality amongst patients receiving ECMO for COVID-19, after adjusting for potential confounding variables and propensity score. In addition, patients receiving corticosteroids with or without other immunomodulators had longer ECMO runs, which has potential implications for resource allocation. While residual confounding likely remains, further studies are required to evaluate the timing of immunomodulators and better understand the possible mechanisms behind this association, including secondary infections.

**Supplementary Information:**

The online version contains supplementary material available at 10.1186/s13613-024-01368-1.

## Introduction

As the coronavirus disease 2019 (COVID-19) pandemic progressed and newer variants of severe acute respiratory syndrome coronavirus 2 (SARS-CoV-2) emerged, the therapeutic options for managing COVID-19 evolved as well. Therapies such as corticosteroids, and other immunomodulators including interleukin-6 antagonists, baricitinib, casirivimab and imdevimab have shown a significant mortality benefit in certain patients receiving conventional oxygen therapy for COVID-19 [1–3].

COVID-19-related acute respiratory distress syndrome (ARDS) has steroid-resistant and steroid-responsive phenotypes, which exhibit different clinical patterns and outcomes [4]. However, studies have found that a “cytokine storm” may not apply to patients with critical COVID-19 [5], and that serum pro-inflammatory cytokines and biomarkers are lower than in patients with non-COVID-19 ARDS [6], raising questions as to whether all immunomodulatory therapy is effective in all patients with critical COVID-19 [7]. In refractory COVID-19 related ARDS, extracorporeal membrane oxygenation (ECMO) has been used as a potential support with variable outcomes. More recent data suggest that mortality rates of ECMO increased over the course of the pandemic [8–12]. It is possible that the use of ECMO later on in the pandemic was more specific to patients who developed refractory ARDS resistant to adjunctive therapies. More importantly, meta-analyses investigating these immunomodulatory therapies such as interleukin-6 antagonists and corticosteroids were unable to ascertain whether these were associated with reductions in mortality [2, 13]. Similarly, a recent meta-analysis found a study-level association between the use of corticosteroids and mortality in patients receiving ECMO [8]. Early retrospective analyses from the Extracorporeal Life Support Organisation (ELSO) registry until July, 2020, showed that immunomodulators increased the risk of superinfections and other complications, but did not affect mortality rates [14]. Others have found that immunomodulatory treatment is associated with secondary infections and mortality. However, this has not been confirmed by all available evidence [15, 16]. We conducted a retrospective cohort study of the ELSO registry to understand the impact of immunomodulatory therapy in a subset of patients with very severe COVID-19-related ARDS who received ECMO.

## Methods

### Data source and participants

This cohort study was conducted following approval by the institutional review board of the National University Hospital, Singapore (NHG DSRB 2022/00389). We adhered to the STrengthening the Reporting of OBservational studies in Epidemiology (STROBE) statement. We used the ELSO registry, an international database which collects anonymised retrospective data on patients receiving ECMO from more than 500 active centres globally. All data entered into the ELSO registry are based on a standardised form by site managers who have been trained and certified for data entry. We included adults (≥ 16 years) who received venovenous ECMO for COVID-19 between 1 January, 2020, through 31 December, 2021. Follow-up data were last updated on 26 April, 2022. For patients with more than one run of ECMO, we used the data from their first run of ECMO. We excluded patients who received other modes of ECMO (including venoarterial, venovenoarterial, and others) or were converted from one mode of ECMO to another, patients where the duration between hospital admission and invasive mechanical ventilation was unknown, patients who received ECMO before invasive mechanical ventilation, did not receive invasive mechanical ventilation, or those who did not have sufficient information regarding invasive mechanical ventilation. We divided patients into four groups: patients not receiving any immunomodulators, patients receiving corticosteroids, patients receiving other immunomodulators (at least one of the following: selective interleukin [IL] blocker including IL-1 [anakinra] and IL-6 blockers [tocilizumab, sarilumab], janus-kinase inhibitors, convalescent plasma, and intravenous immunoglobulins), or a combination of corticosteroid and at least one other immunomodulator, either before or during ECMO.

### Outcomes

The primary outcome was survival time up to 90 days from ECMO initiation. We censored patients who were still receiving ECMO at 90 days after initiation regardless of their final outcome. Patients who survived their run of ECMO to hospital discharge were censored at 90 days. Patients who were discharged within 90 days while still receiving ECMO were censored on the date of discharge. Secondary outcomes included the duration of ECMO, length of hospital stay, and complications experienced while receiving ECMO. We specifically looked at the rates of gastrointestinal haemorrhage (defined by the ELSO registry as upper or lower gastrointestinal haemorrhage requiring more than 3 packed red blood cell transfusions per 24 h, and/or endoscopic intervention, and/or haemostatic agent deployment), and secondary infections (defined by the ELSO SARS-CoV-2 Addendum form as the presence of a secondary infection in addition to COVID-19, including bacterial pneumonia, viral co-infection, bloodstream infection, or urinary tract infection).

### Data synthesis

We summarised patient demographics and clinical characteristics using descriptive statistics. For continuous variables, we used the mean ± SD or median and interquartile range (25th and 75th centiles), whichever was more appropriate. For numerical variables, we reported the counts and percentage. A multinomial regression was used to calculate the propensity score of receiving different treatment strategies (namely – corticosteroids alone, other immunomodulators alone, and a combination of corticosteroids and other immunomodulators), while a logistic regression was used to calculate the propensity score of receiving corticosteroids. After screening for potential predictors, we used a backward model selection method to determine the final model from the following variables: demographics (the presence of cancer, immunocompromised state, diabetes, chronic renal insufficiency, obesity, hypertension, pregnancy, asthma, chronic lung disease excluding asthma, chronic heart disease and frailty), the year in which ECMO was initiated, clinical characteristics (secondary infection, pre-intubation respiratory support, the time between invasive mechanical ventilation and initiation of ECMO, the presence of a codiagnosis defined by the ELSO SARS-CoV-2 Addendum form), and other therapeutic agents (anticoagulation, and other medications including intravenous bicarbonate, epoprostenol, narcotics, neuromuscular blockers, and trisaminomethane).

We compared the survival time up to 90 days between the four groups using a Cox proportional hazards regression model. In the regression models, we adjusted for the propensity score and other potential prognostic factors including age, gender, other coexisting conditions (cancer, pregnancy, immunocompromised, diabetes, chronic lung disease, chronic renal insufficiency, frailty, and obesity), centre size (more than 30 runs of ECMO annually), the time between invasive mechanical ventilation and initiation of ECMO, and the duration of ECMO. We used a quadratic regression to model the duration of ECMO. We considered p-values < 0.05 to be statistically significant.

### Post-hoc analyses

We did several post-hoc sensitivity analyses. First, we censored patient outcomes at 180 days in order to assess the association between immunomodulators with survival time over a longer follow-up horizon. Second, we limited our analysis to patients receiving ECMO for pulmonary support (defined by the ELSO registry as support for respiratory failure by providing gas exchange support). Third, we limited our analysis to patients receiving prone positioning prior to ECMO. We also did a post-hoc subgroup analysis stratifying the cohort based on the year they received ECMO (2020 and 2021), introducing an interaction term between the intervention (the receipt of each immunomodulator) and the year, in order to investigate whether the association between immunomodulators and survival time up to 90 days evolved over time. In view of multiple analyses, we applied a Bonferroni correction, and adjusted the p-value for significance in this subgroup analysis accordingly (threshold for significance = 0.05/3 = 0.0167).

### Role of the funding source

There was no funding source for this study.

## Results

**Fig. 1:**
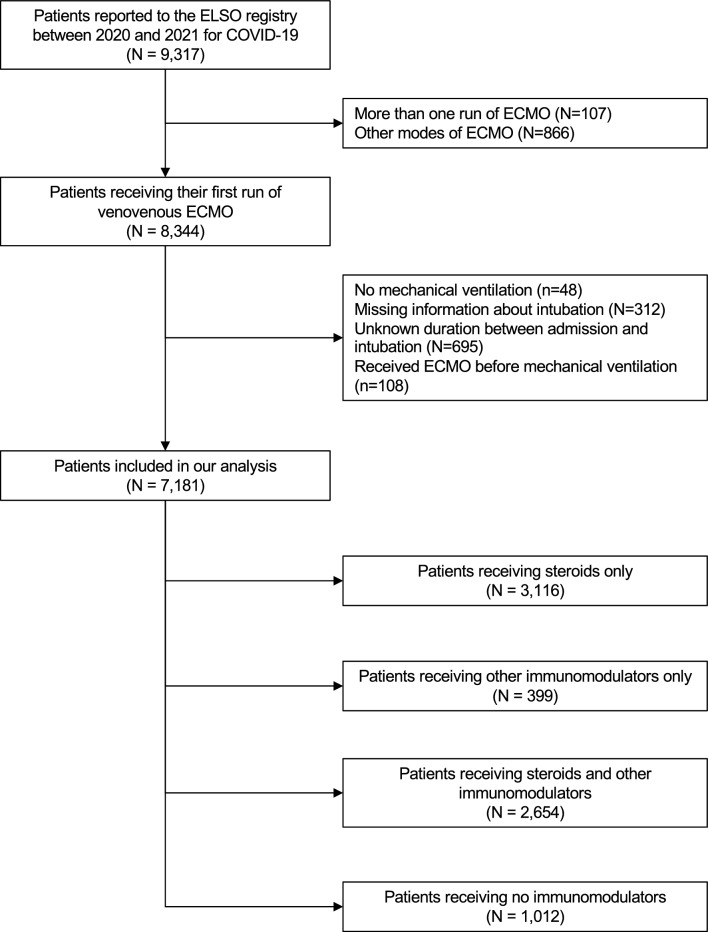
Study inclusion flow diagram

Between 1 January, 2020, and 31 December, 2021, 9317 patients receiving ECMO for COVID-19 were reported to the ELSO registry. After excluding patients who did not meet our inclusion criteria, we included 7181 patients in our analysis (Fig. 1). The mean age was 47.1 ± 11.8 years, and there were no substantial differences between patients in each treatment strategy. The majority of patients were male, and had at least one comorbidity. The most common comorbidities reported were obesity, hypertension, diabetes, and asthma. The median time from endotracheal intubation to ECMO initiation was 74 h in the immunomodulators group, and 93 h for those who did not receive immunomodulators. A small proportion of patients (< 3%) suffered from a cardiac arrest before ECMO. Table 1 summarises the demographic and pre-ECMO factors, while Table S1 presents the haemodynamic, biochemical and ventilator settings both before initiation of ECMO. Patients receiving immunomodulators were more likely to receive vasopressin, narcotics, neuromuscular blockers, inhaled epoprostenol, nitric oxide and intravenous sodium bicarbonate. Table S2 presents the ECMO characteristics after ECMO initiation, and Table S3 presents the ECMO support type and adjunctive therapies used.

**Table 1:** Demographic and pre-ECMO factors

	Steroids only		Other immunomodulators only	Steroids and other immunomodulators	No immunomodulators
Year of ECMO					
* 2020*	1252 (40.1%)		327 (82.0%)	1285 (48.4%)	713 (70.5%)
* 2021*	1864 (59.9%)		72 (18.0%)	1369 (51.6%)	299 (29.5%)
*Age (years)*	46.7 ± 11.7		48.1 ± 11.9	47.3 ± 11.8	47.5 ± 12.0
*Sex*					
Male	2120 (68.1%)		294 (73.7%)	1880 (70.8%)	757 (74.8%)
Female	995 (31.9%)		105 (26.3%)	774 (29.2%)	255 (25.2%)
*Comorbidities*		2427 (77.9%)	308 (77.2%)	2145 (80.8%)	692 (68.4%)
Cancer		40 (1.3%)	7 (1.8%)	49 (1.8%)	10 (1.0%)
Pregnancy		168 (5.4%)	11 (2.8%)	80 (3.0%)	25 (2.5%)
Immunocompromised		109 (3.5%)	7 (1.8%)	113 (4.3%)	27 (2.7%)
Chronic heart disease		104 (3.3%)	4 (1.0%)	77 (2.9%)	24 (2.4%)
Diabetes		780 (25.0%)	121 (30.3%)	747 (28.1%)	239 (23.6%)
Chronic lung disease		118 (3.8%)	14 (3.5%)	100 (3.8%)	25 (2.5%)
Chronic renal insufficiency		88 (2.8%)	17 (4.3%)	79 (3.0%)	19 (1.9%)
Frailty		19 (0.6%)	6 (1.5%)	22 (0.8%)	3 (0.3%)
Obesity		1676 (53.8%)	204 (51.1%)	1570 (59.2%)	459 (45.4%)
Asthma		364 (11.7%)	46 (1.5%)	34 (11.8%)	83 (8.2%)
Hypertension		1005 (32.3%)	134 (33.6%)	989 (37.3%)	258 (25.5%)
Intubation					
* Yes*					
* Pre-existing tracheostomy*		3104	399	2653	1007
* Yes*		12	0	1	5
*Intubation before admission*		1700 (54.6%)	222 (55.6%)	1549 (58.4%)	572 (56.5%)
*Time from admission to intubation (hours)*		− 6.5 (− 75.0 to 71.75)	− 6 (− 69 to 56)	− 10 (− 72.5 to 70)	− 8 (− 90 to 32)
*Time from intubation to ECMO (hours)*		77 (23–148)	74 (28–146)	70 (22.8–140)	93 (30–153)
*Pre-ECMO cardiac arrest*		89 (2.9%)	8 (2.0%)	76 (2.9%)	26 (2.6%)

ECMO: extracorporeal membrane oxygenation

**Fig. 2:**
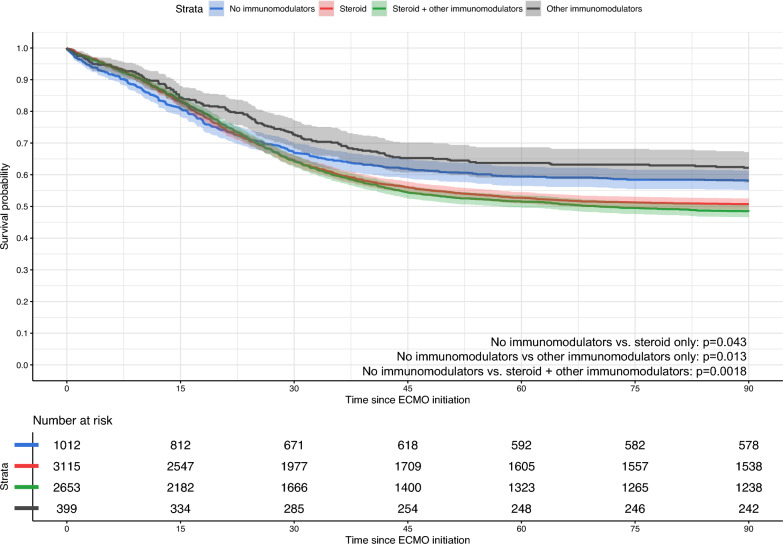
Unadjusted Kaplan–Meier survival curves up to 90 days for patients not receiving immunomodulators, receiving other immunomodulators only, receiving steroids only, and receiving both steroids and other immunomodulators

**Fig. 3:**
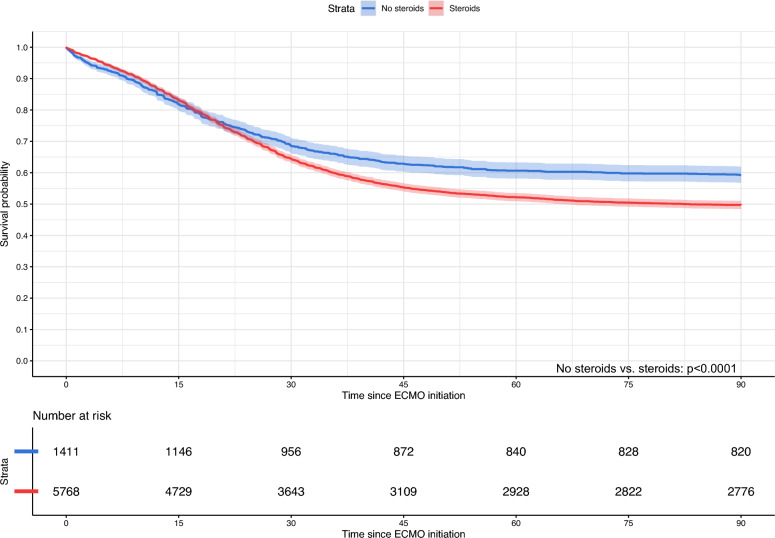
Unadjusted Kaplan–Meier survival curves up to 90 days for patients receiving and not receiving corticosteroids

Of the 7181 patients, 7001 patients had a final outcome; the remaining 180 patients were transferred to another centre or facility while receiving ECMO. 3532 patients died; 3421 died within 90 days after the initiation of ECMO. The overall survival at 90 days for patients receiving no immunomodulators was 58.1% (95%-CI 55.1–61.2%), 50.7% (95%-CI 49.0–52.5%) for those receiving only corticosteroids, 62.2% (95%-CI: 57.4%-67.0%) for those receiving only other immunomodulators, and 48.5% (95%-CI 46.7–50.4%) for those receiving steroids and other immunomodulators. Patients receiving either only corticosteroids (HR: 1.13, 95%-CI 1.01–1.28) or corticosteroids with other immunomodulators (HR: 1.21, 95%-CI: 1.07–1.36) had a significantly lower probability of longer survival time However, patients receiving other immunomodulators without corticosteroids had a higher probability of longer survival time (HR: 0.79, 95%-CI: 0.66–0.96). These results were concordant across sensitivity analyses modifying the maximum follow-up time to 180 days, and when limited to a population receiving VV ECMO for respiratory support or in patients who received prone positioning prior to ECMO (Table S4). When compared to patients who did not receive corticosteroids, patients who received corticosteroids (regardless of other immunomodulators) had a higher hazard of mortality (HR: 1.25, 95%-CI: 1.14–1.39). Table 2 summarises the survival outcomes of patients by immunomodulator treatment groups before or during ECMO. Figure 2 presents Kaplan–Meier curves for patients by treatment groups, while Fig. 3 presents the Kaplan–Meier curves for patients receiving and not receiving corticosteroids.

**Table 2:** Outcomes of patients receiving various immunomodulator treatment strategies while receiving ECMO

Overall cohort				
Treatment strategy	Overall survival		Cox regression model	
	OS (%)	95%-CI	HR	95%-CI
No immunomodulators	58.1	55.1–61.2	Reference	
Corticosteroids only	50.7	49.0–52.5	1.13	1.01–1.28
Other immunomodulators only	62.2	57.4–67.0	0.79	0.66–0.96
Corticosteroids and other immunomodulators	48.5	46.7–50.4	1.21	1.07–1.36

*CI* confidence interval, *HR* hazards ratio, *OS* overall survival

The median duration of ECMO for all patients was 18 (IQR 9–31) days, and the median length of hospital stay was 33 (20–52) days. Patients receiving steroids with and without other immunomodulators had longer durations of ECMO. However, patients receiving other immunomodulators without steroids tended to have longer stays in-hospital. The rates of gastrointestinal haemorrhage did not significantly differ across all groups receiving various combinations of immunomodulators when compared to patients without immunomodulators. However, across all treatment strategies, patients receiving immunomodulators had an increased odds of secondary infections (no immunomodulators: 32.4%, steroids only: 52.5%, other immunomodulators only: 56.3%, steroids and other immunomodulators: 43.1%). Table 3 presents the results of the analysis of all groups of patients with regards to complications.

**Table 3:** Complications of patients while receiving ECMO, stratified by immunomodulator treatment strategy

	Steroids only	Other immunomodulators only	Steroids and other immunomodulators	No immunomodulators
Gastrointestinal bleeding				
Events per 1000 h of ECMO	0.14	0.17	0.11	0.13
Odds ratio	1.16 (0.86–1.58)	1.30 (0.96–1.77)	0.92 (0.55–1.54)	Ref
Secondary infections				
Event count	52.5%	56.3%	43.1%	32.4%
Odds ratio	2.24 (1.93–2.60)	2.53 (2.17–2.96)	1.55 (1.22–1.97)	Ref

*CI* confidence interval, *ECMO* extracorporeal membrane oxygenation

We did a post-hoc analysis stratifying the association between immunomodulators and survival time based on the year ECMO was received (2020 vs. 2021). While the association between immunomodulators and mortality increased from 2020 and 2021 across all groups, there were no interaction effects between 2020 and 2021 (overall interaction p-value: 0.093, individual interaction p-values all > 0.0167; individual HRs and p-value for each immunomodulator are summarised in Table S5).

## Discussion

In this cohort of 7181 patients, our propensity-adjusted analysis found that the use of steroids with and without other immunomodulators before or during ECMO was significantly associated with mortality amongst patients receiving ECMO for COVID-19. However, patients receiving only other immunomodulators had lower risk of death. Additionally, immunomodulators were associated with secondary infections in patients with COVID-19 receiving ECMO.

The current evidence base for the use of immunomodulatory treatment for patients receiving ECMO remains unclear. While some studies have suggested that the use of corticosteroids was associated with lower 90-day mortality in experienced ECMO centres [18], other studies within and without COVID-19 have shown that the use of immunomodulatory treatment was associated with increased mortality [19, 20]. This stands in contrast to other studies which suggests that the use of immunomodulatory treatment in severe-to-critical COVID-19 significantly reduces mortality [3]. However, it is likely that immunomodulatory treatment remains effective for patients with severe COVID-19 [1, 3, 7, 13, 17], and may only be harmful in a select subgroup of patients who have critical ARDS refractory to conventional and adjunctive therapies, who had a higher baseline risk of worse outcomes.

It is possible that many patients received immunomodulators prior to ECMO initiation and may represent a cohort that had failed early immunomodulatory and other supportive therapies with severe disease progression and consequent poor outcomes. Equally, it is possible that the risk tolerance for these therapies in critically ill patients receiving ECMO for COVID-19 may be low. Our analysis indicates that the complications such as secondary infections may outweigh the benefits in these patients with complex pathologies requiring prolonged ECMO support. However, these findings should be interpreted in the context of an evolving pandemic with multiple factors such as evolving viral strains, variable resource availability and vaccination uptake, adoption of multiple immunomodulatory drugs in early disease leading to greater degree of immunosuppression before ECMO initiation, and increasing uptake of less invasive respiratory supports as the pandemic progressed, which potentially delayed ECMO initiation. [10, 11]

With the increased use of immunomodulatory treatment over time, it is possible that there exists a bias in the selection of patients who receive ECMO [8]. Prior studies have shown that there exist steroid-resistant and steroid-responsive phenotypes in COVID-19, with each phenotype displaying a divergent clinical course [4]. Given that immunomodulatory treatment only featured later on in the pandemic, patients who would have required ECMO earlier in the pandemic might have recovered and eventually not required ECMO. The patients who then required ECMO later on in the pandemic might demonstrate the steroid-resistant phenotype consistent with hypoinflammatory ARDS associated with superinfection, receive potentially inappropriate steroid therapy, and subsequently progress with illness severity and require ECMO. In the post-hoc subgroup analysis stratifying the associations by year of ECMO, we found that the associations between immunomodulators and mortality generally increased from 2020 to 2021; nonetheless this did not reach statistical significance. The use of immunomodulators also doubled the risk of secondary infections while receiving ECMO in our cohort after adjusting for the duration of ECMO, a fact that has been observed both in patients receiving and not receiving ECMO [14, 15]. It is interesting to note that patients receiving a combination of steroids and other immunomodulators had a relatively lower odds of secondary infections when compared to those receiving steroids or immunomodulators alone. This may be explained by immortal time bias – that patients must first survive for sufficiently long before they suffer from a potential complication such as secondary infections. In order to account for this, we adjusted for the duration of ECMO.

Studies have found that secondary infections and superinfections in patients with COVID-19 are associated with higher mortality [15, 21, 22]. However, meta-analyses of randomised controlled trials investigating the use of immunomodulators in COVID-19 have suggested that these therapies are not associated with an increased risk of secondary infections [23, 24], although there remains limited evidence for the efficacy of these interventions in patients receiving ECMO. It is possible that these adverse events might not always be captured by initial randomised controlled trials because they might not have sufficient power to detect rare adverse events [25], particularly when only a subset of patients who receive these immunomodulators required mechanical ventilation and/or ECMO. The ELSO registry collects a larger number of patients who receive ECMO for COVID-19 compared to these trials, and reports more granular patient-level data with regards to outcomes such as adverse events and complications.

There are two main strengths of our study. First, our study is based on an international registry that spans more than 500 centres globally. The ELSO registry also provides detailed patient-level data about demographics, clinical characteristics, treatments received, and outcomes and is subject to rigorous quality improvement measures. The rapid introduction of a detailed COVID-19 addendum into the registry early in the pandemic resulted in more granular and richer data for analysis in a quick and timely fashion. The large sample size increased the precision of our results, and adequately powered our analysis to detect any differences in mortality amongst patients who did or did not receive immunomodulators. Second, we were able to account for confounding through multivariable regression and propensity score adjustment.

There are also several limitations of our study. First, our study is based on retrospective data reported to the ELSO registry. Although propensity-score weighting methods are able to mitigate this limitation, there are other possible residual confounders which may not have been reported, or accounted for, including both patient- and hospital-level factors. In addition, with a wide range of medications being grouped as “other immunomodulators”, this introduced heterogeneity into the analysis, and we cannot the exclude the possibility that certain specific medications may be associated with benefits, while others with harm. Second, the data submitted to the ELSO registry is submitted voluntarily and is not externally validated. Third, the data reported to the ELSO registry did not elaborate on the exact timing (on which day of or prior to ECMO), nor the dosing (e.g. high-dose or low-dose corticosteroids) of immunomodulators administered. This might potentially be an important factor in the patient’s prognosis [26], and we were unable to evaluate any potential benefits of late administration of immunomodulators for reparative stages of COVID-19-related ARDS. In addition, our analysis was not designed not powered to assess differences between different types of immunomodulators or treatment regimens. Fourth, immunomodulators were largely introduced as a treatment for select COVID-19 patients towards the end of 2020 and in 2021. Longitudinally, other possible factors that might confound mortality and patient selection for ECMO include virus variants, type and dose of immunomodulator used, the more common and prolonged use of non-invasive ventilation, and changes in patient selection based on local resource availability changes. While we have attempted to account for this by adjusting for the year of ECMO (2020 and 2021) as well as centre volumes in our propensity score models, this may not fully account for the time-varying covariates other than the use of immunomodulators which might affect patient outcomes.

## Conclusion

In conclusion, our review of the ELSO registry found that steroids with or without other immunomodulators were associated with longer ECMO runs, and higher mortality. Patients receiving other immunomodulators without steroids had a lower risk of death. Immunomodulators (regardless of the exact medication) were associated with secondary infections. It is plausible that patients who receive ECMO for COVID-19-related ARDS are refractory to adjuvant therapies and continuing immunomodulators during ECMO may contribute to more complications and poorer outcomes. While immunomodulatory treatment may remain effective for most patients with severe COVID-19, it may have been harmful in a subgroup of patients who have COVID-19 refractory to all known therapy. Further studies are required to evaluate for the timing of immunomodulators and their effects on mortality, as well as better understand the possible mechanisms for this increase in mortality. These findings have potential implications in future viral pandemics.

### Supplementary Information

Supplementary Material 1

## Data Availability

The data dictionary and ELSO policies are available online. The individual participant data collected for this study are available as a limited dataset to member centres following approval from the ELSO Scientific Oversight Committee, but it is not publicly available. ELSO Scientific Oversight Committee approved data requests and the data of data release for research are listed online at https://www.elso.org/Registry/ApprovedDataRequests.aspx
