# Supplementary material for: Immunomodulators in patients receiving extracorporeal membrane oxygenation for COVID-19: a propensity-score adjusted analysis of the ELSO registry
Source: Ann Intensive Care. 2024 Aug 20;14:128. doi: 10.1186/s13613-024-01368-1 (PMC11336150; doi:10.1186/s13613-024-01368-1)
Supplement: Supplementary file 1 — Supplementary Material 1 [file 13613_2024_1368_MOESM1_ESM.docx]

**Immunomodulators in patients receiving extracorporeal membrane oxygenation for COVID-19: a propensity-score adjusted analysis of the ELSO registry.**

*Ryan Ruiyang Ling MBBS,^1^ (ORCID: 0000-0002-8335-7640),

*Kollengode Ramanathan MD, ^1,2^ (ORCID: 0000-0003-1822-9455),

Liang Shen PhD, ^3^

Ryan P Barbaro MD,^4^ (ORCID: 0000-0002-3645-0359),

Prof Kiran Shekar PhD,^5,6^ (ORCID: 0000-0002-1239-7514),

Prof Daniel Brodie MD, ^7^ (ORCID: 0000-0002-0813-3145),

Prof Graeme MacLaren MSc,^1,2^ (ORCID: 0000-0002-1307-4274).

_________________________________________________________________________________

*Joint first authors

^1^ Yong Loo Lin School of Medicine, National University of Singapore, National University Health System, Singapore

^2^ Cardiothoracic Intensive Care Unit, National University Heart Centre, National University Hospital, National University Health System, Singapore

^3^ Biostatistics Unit, Yong Loo Lin School of Medicine, National University of Singapore, National University Health System, Singapore

^4^ Division of Paediatrics Critical Care Medicine, University of Michigan, Ann Arbor; Child Health Evaluation and Research Center, University of Michigan, Ann Arbor, Michigan, USA

^5^ Adult Intensive Care Services, Prince Charles Hospital, Brisbane, Queensland, Australia

^6^ Queensland University of Technology, Brisbane; University of Queensland, Brisbane and Bond University, Gold Coast, Queensland, Australia

^7^ Division of Pulmonary and Critical Care Medicine, Department of Medicine, The Johns Hopkins University School of Medicine, Baltimore, MD, USA

Corresponding author:

Kollengode Ramanathan, MD

Email: [surrkr@nus.edu.sg](mailto:surrkr@nus.edu.sg)

Cardiothoracic Intensive Care Unit, National University Heart Centre, National University Hospital, National University Health System.

Level 9, 1E Kent Ridge Road, Singapore 119228

**Table of contents**

[**Table S1.** Haemodynamic, biochemical, and ventilator settings pre-ECMO 3](#_Toc171279658)

[**Table S2.** Haemodynamic, biochemical, and ventilator settings 24 hours after ECMO initiation 4](#_Toc171279659)

[**Table S3.** ECMO characteristics and adjunctive therapies. 5](#_Toc171279660)

[**Table S4.** Post-hoc sensitivity analyses 7](#_Toc171279661)

[**Table S5.** Results of post-hoc subgroup analysis 7](#_Toc171279662)

# **Table S1.** Haemodynamic, biochemical, and ventilator settings pre-ECMO

|  | **Pre-ECMO** | | | |
| --- | --- | --- | --- | --- |
|  | Steroids only | Other immunomodulators only | Steroids and other immunomodulators | No immunomodulators |
| **Haemodynamics** | | | | |
| SBP (mmHg) | 121.2 ± 23.5 | 119.1 ± 23.3 | 119.8 ± 23.7 | 119.0 ± 23.9 |
| DBP (mmHg) | 65.1 ± 13.0 | 64.1 ± 12.7 | 64.4 ± 12.6 | 64.1 ± 13.1 |
| MAP (mmHg) | 79.2 ± 11.9 | 78.1 ± 11.1 | 78.9 ± 11.9 | 79.6 ± 12.7 |
| **Biochemical data** | | | | |
| pH | 7.29 ± 0.12 | 7.27 ± 0.11 | 7.28 ± 0.12 | 7.28 ± 0.12 |
| PaCO_2_ | 65.1 ± 22.5 | 65.7 ± 25.4 | 66.1 ± 24.8 | 65.1 ± 21.8 |
| PaO_2_ | 73.0 ± 41.2 | 81.6 ± 72.0 | 78.3 ± 58.1 | 73.2 ± 34.2 |
| HCO_3_ | 29.1 ± 6.9 | 28.4 ± 6.2 | 29.2 ± 6.9 | 28.4 ± 6.9 |
| SaO_2_ | 88.0 ± 9.2 | 89.4 ± 7. | 88.2 ± 10.5 | 88.6 ± 9.0 |
| PaO_2_/FiO_2_ | 79.0 ± 45.3 | 89.7 ± 86.7 | 84.6 ± 74.0 | 81.2 ± 43.2 |
| **Ventilatory settings** | | | | |
| Rate | 26.0 ± 6.7 | 26.3 ± 6.3 | 26.2 ± 6.6 | 25.6 ± 7.0 |
| FiO_2_ | 94.3 ± 11.8 | 94.6 ± 12.1 | 94.5 ± 11.7 | 93.2 ± 12.9 |
| PIP | 33.7 ± 6.7 | 33.8 ± 6.4 | 34.2 ± 6.8 | 32.9 ± 7.3 |
| PEEP | 13.1 ± 4.1 | 13.5 ± 4.0 | 14.4 ± 4.3 | 13.6 ± 3.9 |
| Mean AP | 21.9 ± 5.4 | 22.6 ± 5.3 | 22.0 ± 5.0 | 22.3 ± 5.5 |
|  | **Day of intubation** | | | |
|  | Steroids only | Other immunomodulators only | Steroids and other immunomodulators | No immunomodulators |
| C-reactive protein | 101.0 (21.0-263.5) | 37.28 (16.88-197.13) | 22.7 (8.0-104.35) | 101.0 (21.0 – 263.5) |
| Procalcitonin | 0.37 (0.15-1.23) | 0.52 (0.18-1.79) | 0.34 (0.13-0.99) | 0.58 (0.23 – 1.87) |

Abbreviations: AP: airway pressure, FiO_2_: fraction of inspired oxygen, DBP: diastolic blood pressure, ECMO: extracorporeal membrane oxygenation, HCO_3_: bicarbonate, MAP: mean arterial pressure, PaCO_2_­: partial pressure of carbon dioxide, PaO_2_: partial pressure of oxygen, PEEP: positive end-expiratory pressure, PIP: peak inspiratory pressure, SaO_2_: arterial saturation of oxygen, SBP: systolic blood pressure

# **Table S2.** Haemodynamic, biochemical, and ventilator settings 24 hours after ECMO initiation

|  | **24 hours after ECMO initiation** | | | |
| --- | --- | --- | --- | --- |
|  | Steroids only | Other immunomodulators only | Steroids and other immunomodulators | No immunomodulators |
| **Haemodynamics** | | | | |
| SBP (mmHg) | 119.7 ± 18.7 | 117.7 ± 18.2 | 120.7 ± 19.4 | 119.4 ± 19.0 |
| DBP (mmHg) | 61.8 ± 10.3 | 60.1 ± 10.2 | 61.2 ± 10.6 | 62.5 ± 11.2 |
| MAP (mmHg) | 79.2 ± 11.9 | 78.1 ± 11.1 | 78.9 ± 11.9 | 79.6 ± 12.7 |
| **Biochemical data** | | | | |
| pH | 7.40 ± 0.07 | 7.40 ± 0.06 | 7.30 ± 0.07 | 7.39 ± 0.09 |
| PaCO_2_ | 47.7 ± 14.3 | 48.2 ± 23.9 | 48.2 ± 18.0 | 48.0 ± 14.7 |
| PaO_2_ | 81.2 ± 40.8 | 90.3 ± 75.1 | 87.4 ± 60.6 | 85.6 ± 41.3 |
| HCO_3_ | 28.9 ± 5.5 | 28.2 ± 5.3 | 28.7 ± 5.4 | 28.2 ± 5.8 |
| SaO_2_ | 93.5 ± 4.6 | 93.3 ± 5.1 | 93.5 ± 5.0 | 93.7 ± 4.8 |
| **Ventilatory settings** | | | | |
| Rate | 13.4 ± 5.0 | 14.2 ± 5.6 | 13.7 ± 5.1 | 13.6 ± 5.1 |
| FiO_2_ | 57.1 ± 24.5 | 62.4 ± 25.8 | 58.9 ± 24.5 | 62.3 ± 27.3 |
| PIP | 24.5 ± 5.6 | 25.4 ± 6.3 | 25.5 ± 5.6 | 23.7 ± 5.4 |
| PEEP | 10.6 ± 3.0 | 11.5 ± 3.3 | 10.8 ± 3.0 | 11.2 ± 2.9 |
| Mean AP | 15.6 ± 4.3 | 16.2 ± 4.3 | 16.0 ± 4.2 | 16.1 ± 4.3 |
|  | **24 hours after ECMO initiation** | | | |
|  | Steroids only | Other immunomodulators only | Steroids and other immunomodulators | No immunomodulators |
| C-reactive protein | 158.6 (67.0-261.0) | 116.3 (24.7-270.0) | 69.4 (16.7-170) | 205.6 (84.2-326) |
| Procalcitonin | 0.62 (0.21-2.46) | 0.78 (0.25-2.99) | 0.46 (0.19-1.64) | 1.53 (0.46 – 5.45) |

Abbreviations: AP: airway pressure, FiO_2_: fraction of inspired oxygen, DBP: diastolic blood pressure, ECMO: extracorporeal membrane oxygenation, HCO_3_: bicarbonate, MAP: mean arterial pressure, PaCO_2_­: partial pressure of carbon dioxide, PaO_2_: partial pressure of oxygen, PEEP: positive end-expiratory pressure, PIP: peak inspiratory pressure, SaO_2_: arterial saturation of oxygen, SBP: systolic blood pressure

# **Table S3.** ECMO characteristics and adjunctive therapies.

| ECMO Characteristics | | Steroids only | Other immunomodulators only | Steroids and other immunomodulators | No immunomodulators |
| --- | --- | --- | --- | --- | --- |
| ***ECMO support type*** | |  |  |  |  |
|  | Pulmonary | 3106 (99.7%) | 399 (100.0%) | 2646 (99.7%) | 1008 (99.6%) |
|  | Cardiac | 6 (0.2%) | 0 (0.0%) | 4 (0.2%) | 2 (0.2%) |
|  | ECPR | 4 (0.1%) | 0 (0.0%) | 4 (0.2%) | 2 (0.2%) |
| ***Adjunctive therapies*** | |  |  |  |  |
| ***Any form of vasoactive medications*** | |  |  |  |  |
|  | Norepinephrine | 1590 (51.0%) | 210 (52.6%) | 1392 (52.4%) | 524 (51.8%) |
|  | Epinephrine | 116 (3.7%) | 19 (4.8%) | 100 (3.8%) | 46 (4.5%) |
|  | Vasopressin | 260 (8.3%) | 47 (11.8%) | 295 (11.1%) | 99 (9.8%) |
| ***Any other medications*** | |  |  |  |  |
|  | Other synthetic prostacyclin analogues | 115 (3.7%) | 16 (4.0%) | 79 (3.0%) | 35 (3.5%) |
|  | Narcotics | 2372 (76.1%) | 286 (71.7%) | 2123 (80.0%) | 618 (61.1%) |
|  | Neuromuscular blockers | 2408 (77.3%) | 288 (72.2%) | 2036 (76.7%) | 632 (62.5%) |
|  | IV Sodium bicarbonate | 210 (6.7%) | 31 (7.8%) | 211 (8.0%) | 61 (6.0%) |
|  | THAM | 9 (0.3%) | 0 (0.0%) | 9 (0.3%) | 3 (0.3%) |
| ***Renal, pulmonary, or other support*** | |  |  |  |  |
|  | Prone positioning | 1848 (59.3%) | 238 (59.6%) | 1562 (58.9%) | 565 (55.8%) |
|  | Inhaled epoprostenol | 453 (14.5%) | 91 (22.8%) | 573 (21.6%) | 135 (13.3%) |
|  | Nitric oxide | 417 (13.4%) | 56 (14.0%) | 440 (16.6%) | 110 (10.9%) |
|  | Renal replacement therapy | 156 (5.0%) | 30 (7.5%) | 112 (4.2%) | 81 (8.0%) |
| ***Pre-intubation non-invasive ventilation*** | |  |  |  |  |
|  | CPAP | 527 (96.2%)  N = 548 | 38 (88.4%)  N = 43 | 318 (93.8%)  N = 339 | 120 (97.6%)  N=123 |
|  | BiPAP | 1256 (97.0%)  N = 1295 | 111 (100.0%)  N = 111 | 1269 (98.2%)  N = 1292 | 226 (95.0%)  N = 238 |
|  | HFNC | 1632 (99.0%)  N = 1649 | 214 (99.1%)  N = 216 | 1622 (99.2%)  N = 1635 | 344 (98.0%)  N = 351 |

Abbreviations: BiPAP: bi-level positive airway pressure, CPAP: continuous positive airway pressure ECMO: extracorporeal membrane oxygenation, ECPR: extracorporeal cardiopulmonary resuscitation, HFNC: high-flow nasal cannula, IV: intravenous, THAM: tris(hydroxymethyl)aminomethane

# **Table S4.** Post-hoc sensitivity analyses

| **Intervention** | **Hazard ratio** | | |
| --- | --- | --- | --- |
|  | Censoring at 180 days | Limiting to pulmonary support | Limiting to patients receiving pre-ECMO prone positioning |
| No immunomodulators | Reference | | |
| Steroids only | 1.14 (1.01-1.28) | 1.14 (1.01-1.28) | 1.13 (0.96-1.32) |
| Other immunomodulators only | 0.83 (0.69-0.996) | 0.80 (0.66-0.97) | 0.77 (0.60-0.99) |
| Steroids and other immunomodulators | 1.21 (1.07-1.36) | 1.21 (1.07-1.36) | 1.26 (1.08-1.48) |

**Table S5.** Results of post-hoc subgroup analysis.

| **Intervention** | **Year 2020** | | **Year 2021** | | **Interaction p-value** |
| --- | --- | --- | --- | --- | --- |
|  | HR | CI | HR | CI |  |
| No immunomodulators | Reference group | | | | |
| Steroids only | 1.07 | 0.92-1.24 | 1.25 | 1.01-1.53 | 0.021 |
| Steroids and other immunomodulators | 1.13 | 0.98-1.31 | 1.33 | 1.08-1.65 | 0.020 |
| Other immunomodulators only | 0.72 | 0.58-0.90 | 1.00 | 0.67-1.51 | 0.083 |
